# Development of a feasible and acceptable digital prehabilitation pathway to improve elective surgical outcomes

**DOI:** 10.3389/fdgth.2023.1054894

**Published:** 2023-02-09

**Authors:** Ecushla C. Linedale, Eleanor Bills, Anastasia Dimopoulos, Jackie Yeoh, Mandy Nolan, Vicki Hume, Sharyn Coles, Jane M. Andrews

**Affiliations:** ^1^ Health Translation SA, South Australian Health and Medical Research Institute, Adelaide, Australia; ^2^ Faculty of Health Sciences, School of Medicine, The University of Adelaide, Adelaide, Australia; ^3^ Surgery Program, The Central Adelaide Local Health Network, Adelaide, Australia; ^4^ GP Liaison Unit, The Central Adelaide Local Health Network, Adelaide, Australia; ^5^ Adelaide Primary Health Network, Adelaide, Australia

**Keywords:** prehabilitation, surgical complications, digital pathway, elective surgery, health service improvement

## Abstract

**Objective(s):**

To codesign and assess the feasibility, acceptability, and appropriateness of a hospital-initiated, community delivered approach to health optimization (prehab) prior to planned surgery.

**Design:**

Participatory codesign combined with a prospective, observational cohort study (April–July 2022).

**Setting:**

A large metropolitan tertiary referral service with 2 participating hospitals.

**Participants:**

All people referred for orthopaedic assessment for joint replacement surgery (hip or knee) triaged as category 2 or 3. Exclusions: category 1; no mobile number. Response rate 80%.

**Intervention:**

*My PreHab Program* is a digitally enabled pathway that screens participants for modifiable risk factors for post-operative complications and provides tailored information to enable health optimization prior to surgery with the help of their regular doctor.

**Outcome measures:**

Acceptability, feasibility, appropriateness, and engagement with the program.

**Results:**

36/45 (80%) registered for the program (ages 45–85 yrs.), completed the health-screening survey and had ≥1 modifiable risk factor. Eighteen responded to the consumer experience questionnaire: 11 had already seen or scheduled an appointment with their General Practitioner and 5 planned to. 10 had commenced prehab and, 7 planned to. Half indicated they were likely (*n* = 7) or very likely (*n* = 2) to recommend *My PreHab Program* to others. The *My PreHab Program* scored an average 3.4 (SD 0.78) for acceptability, 3.5 (SD 0.62) for appropriateness, and 3.6 (SD 0.61) for feasibility, out of a score of 5.

**Conclusion(s):**

This digitally delivered intervention is acceptable, appropriate, and feasible to support a hospital-initiated, community-based prehab program.

## 1. Introduction

Surgery is a critical component of healthcare systems around the world (1, 2). Yet with Increasing constraints on resources, an ageing population and growing burden of chronic disease, surgical facilities worldwide face increasing challenges in providing accessible, affordable, and safe elective surgery. This capacity problem is contributed to by potentially preventable post-operative complications.

Post-operative complications are common (affecting 20% of surgical episodes) and a significant cause of morbidity and mortality (3, 4). Post-operative complications affect individuals by poorer health outcomes and higher risk of death and have negative effects on the healthcare system *via* increased length of stay (more than double) (3) and greater rate of re-admission (3, 4) thus reducing ‘bed availability’ and prolonging surgical wait times. All of these result in significant costs and delays to the health system and community whose taxes fund the public health system (5).

These complications are increasing at a rate of 10% annually and have recently been described as a “hidden pandemic” (5, 6). There is an immediate imperative to slow this increasing rate and provide safer, high-value care within constrained health system budgets. Often, the focus of attention is on reducing variation in clinical performance (clinicians and hospitals), however another opportunity is to reduce risk before the consumers arrive - even well before their surgery is scheduled. This opportunity may have been previously overlooked or ignored as it crosses the community-hospital boundary and might be seen as ‘too hard’.

Prehabilitation (prehab) is an intervention that aims to identify and address modifiable risk factors (smoking, anaemia), improving aerobic capacity, nutritional balance, and psychological status before surgery (7). Small, randomised control trials of limited/single components of prehab suggest a large reduction in complications [41% (95% confidence interval [CI], 15–59, *P = .*01) for smoking cessation] (8) for prevention of postoperative complications., and good return on investment with shorter length of stay, lower hospital costs and reduced healthcare utilisation post-discharge (7). However, no-one has reported on the feasibility, acceptability, and/or outcomes of a ‘global’ prehab approach, rolling all modifiable risk factors into a proactive screening approach.

**Figure 1:**
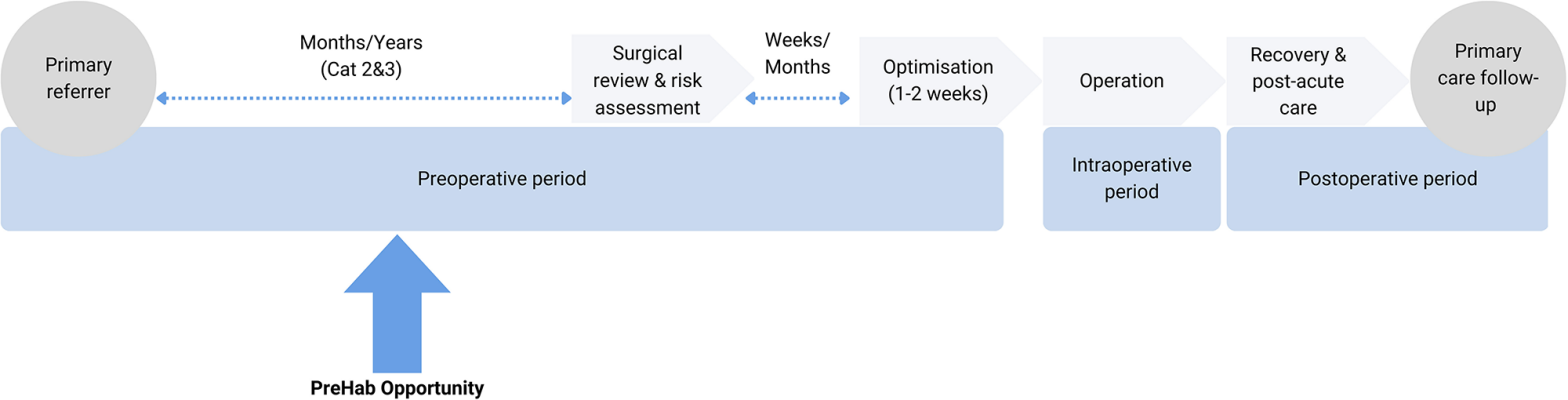
Perioperative timeline (adapted from ANZCA 2019) (10).

Current systems do not routinely ensure preoperative optimization occurs. Currently, pre-operative optimization occurs within a specialized preoperative anesthetic clinic (when it does occur), generally within 1–2 weeks before surgery. Whilst there is value in this, many aspects of optimization would deliver greater gains if identified and addressed over a longer time. For example, smoking cessation is best 4 or more weeks prior to surgery (9) and more weight loss is likely to be achieved over a longer period. This imperative to push back preoperative surgical optimization timelines led us to propose that prehab (getting healthy before surgery) should commence at the time of referral (Figure 1) for consideration of non-emergency elective surgery so optimization(s) can commence earlier, and perhaps even negate the need for surgery.

This study aims to describe the codesign and evaluation of a hospital initiated, community delivered approach to ‘global’ prehab that included proactive digital screening, tailored, personalised advice and ensuring good quality resources were available for patient self-service and community healthcare provider support. The concept to be tested was that this approach would turn the often-long wait times for people awaiting surgical assessment (consumers) into an opportunity for active engagement in preparing for surgery. Specifically, we sought to investigate whether this approach was acceptable and feasible to people awaiting surgical assessment in the public health system (herein termed consumers) and their primary healthcare providers and would lead to engagement in activity to optimise health.

The work builds on our previous findings that:

a) People on specialist outpatient waitlists actively engage in screening and hospital directed self-management (IBS) (11)
b) Consumers and their regular doctors already use decision support tools (11, 12)
c) Digital technology is a feasible, acceptable and effective mode of facilitating the above (13).

We describe here the codesign and initial user testing of the *My PreHab Program*, and assess its feasibility, acceptability, and appropriateness for consumers referred for orthopaedic assessment for total hip or knee replacement surgery. The *My Prehab Program* is a hospital-initiated, community delivered approach to health optimization (prehab) prior to planned surgery. This cohort of orthopaedic assessment referrals was chosen as the initial target due to the lengthy wait times they often experience.

This study is nested within a multi-year clinical effectiveness study – the results of which will be published in the future.

## 2. Method

### 2.1. Study design and approach

The project was comprised of three integrated phases:

1) Collaborative codesign of *My PreHab* website
2) Understanding the needs and perspectives of consumers and primary healthcare providers
3) Design and testing of *My PreHab Pathway* in Orthopaedic Surgery.

Together, the *My PreHab* website and *My PreHab Pathway* make up the *My PreHab Program*.

A collaborative stakeholder approach was used to ensure all relevant stakeholders were meaningfully engaged throughout. The project was led by a multi-professional steering group with extensive experience across many healthcare settings and sites.

**Figure 2:**
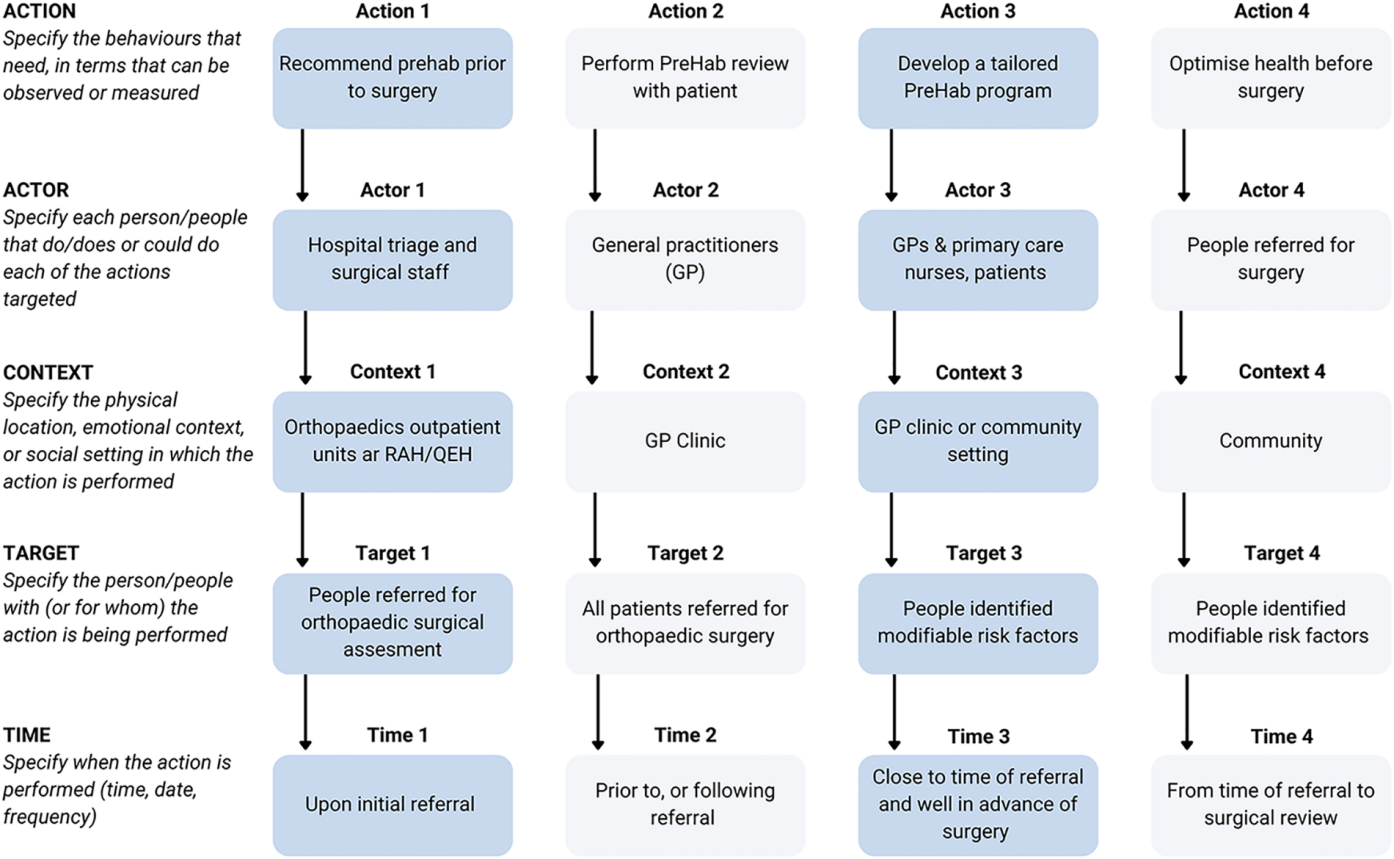
Use of the AACTT framework (14) to map the desired behavioral change objectives of the *My PreHab Program*.

**Figure 3:**
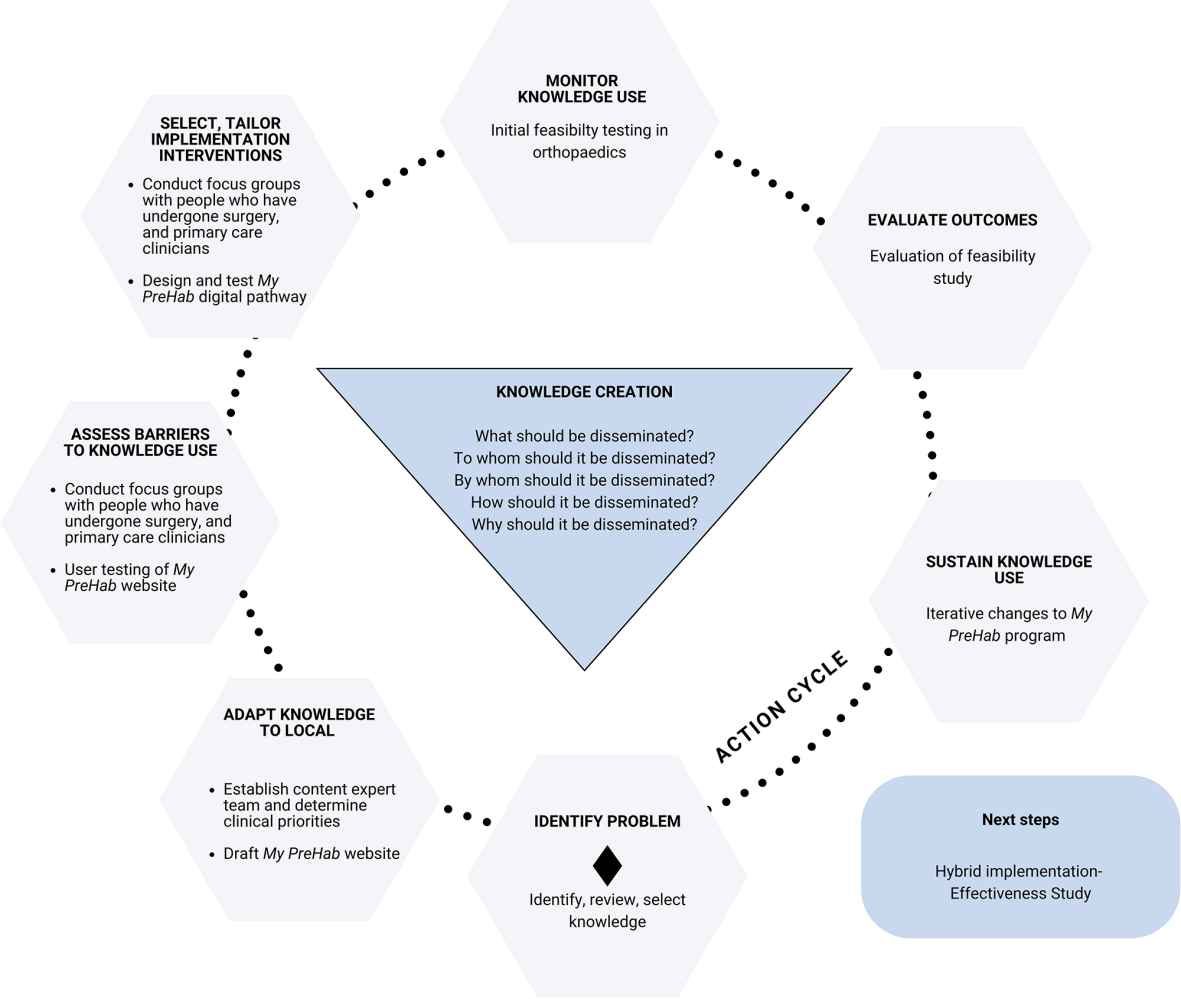
Use of the KTA framework (15) to guide the development and implementation of the *My PreHab Program*.

The Action, Actor, Context, Target, Time (AACTT) Framework (14) was used to identify the desired behavioural changes required to improve consumers health preoperatively, and inform stakeholder engagement (Figure 2). The Knowledge to Action (KTA) framework (15) was used to conceptualise and guide the process of moving evidence into practice which we aimed to achieve in this study (Figure 3).

**Figure 4:**
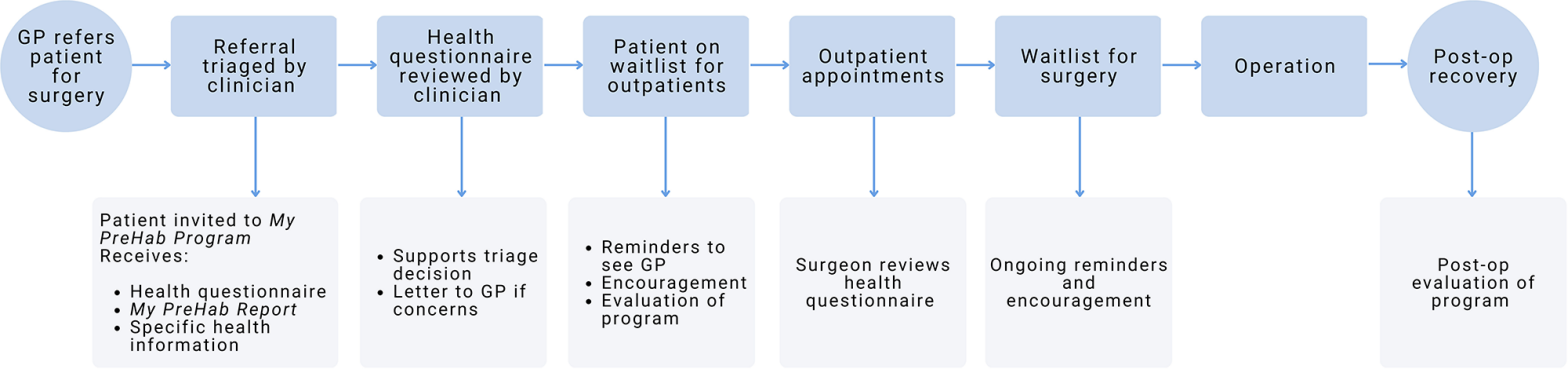
*My PreHab Pathway*.

### 2.2. Phase 1: Collaborative codesign of *My PreHab* website

The *My PreHab* content development group comprised a team of experts in clinical care, covering the known modifiable risk factors: smoking cessation, diabetes management, anaemia +/- iron deficiency, emotional wellbeing, frailty, pain management, alcohol-drugs-and-medication management, activity and exercise, nutrition, and weight optimisation. The group included a Central Adelaide Local Health Network (CALHN) Surgical Program Medical Lead (JMA; FRACP, Gastroenterologist), a health service researcher, orthopaedic and general surgeons, orthopaedics and diabetes nurses, chronic pain specialists (anaesthetists & psychologists), allied health specialists (nutrition and dietetics, psychology, exercise physiology, occupational therapy and physiotherapy), general practice and intermediate care clinicians and a consumer representative. The content development group undertook an iterative process of mapping and selecting publicly available evidence-based resources already vetted at a national level and drafting summary information about each risk factor for consumers. Content consensus was achieved through regular content expert group meetings and website review. Additional relevant and/or interested clinicians in these fields were also invited to provide input. The draft website was made available to General Practitioners (GPs), practice nurses and consumers who participated in Phase 2, and feedback was used to inform the final round of content change prior to user testing as a component of the *My PreHab Program*.

### 2.3. Phase 2: Understanding the needs and perspectives of GPs and consumers

Two focus groups were conducted with consumers and primary care providers to fully understand their perspectives and needs and to identify barriers and enablers for prehabilitation. The Theoretical Domains Framework (16) was used to develop flexible sets of focus group questions to ensure all major barriers and enablers were identified and to avoid confirmation bias (Supplementary Appendices A, B).

People who had undergone surgery in South Australia within the past two years were recruited through the Health Translation SA Consumer Registry. The 2 h focus group with seven consumers was conducted in person. Primary healthcare providers (GPs and Practice Nurses) were recruited *via* the Adelaide Primary Health Network newsletter and Health Translation SA's social media network. A 2 h online focus group was conducted with 15 primary healthcare providers. The CALHN Surgical lead (JMA) presented information about the issue of post-operative complications from the perspective of the hospital system and the proposed role of prehabilitation. An open discussion followed (guided by Supplementary Appendix A). Feedback was also sought on the website features and content.

Data were analysed from the focus group using a pragmatic mind mapping approach with a summary of main themes documented. These were used to inform design changes to the *My PreHab* website and design considerations for the *My PreHab* digital pathway.

### 2.4. Phase 3: Design and testing of *My PreHab Program* in orthopaedic surgery

*Codesign* workshops were held with the Orthopaedic Nurse Consultant and Practitioner at the Royal Adelaide Hospital and The Queen Elizabeth Hospital. Together, the current clinical pathways were mapped for consumers referred for orthopaedic assessment for joint replacement surgery (hip or knee) and ensured workflow integration of the digital *My PreHab Pathway*. The digitally enabled *My PreHab Pathway* was developed on the Personify Care platform (www.personifycare.com).

Two rounds of testing were conducted with consumers triaged to a category two or three on the outpatient waitlist. Consumers whose referrals were triaged as ‘urgent’ (category 1) and those without a personal mobile contact number were excluded. Initial user-testing to assess the functionality of the digital pathway was conducted in 30 referred consumers in orthopaedics (15 consumers from each site). Measures focussed on the number of consumers who engaged with each step of the *My PreHab Pathway*. Non-responders were phoned by the Orthopaedic Nurse Consultant and Practitioner to ask them to complete the registration. Changes were made to the pathway based on the user-testing results and the pathway went ‘live’ in Orthopaedics for Beta-testing in every patient referred during July 2022. Additional Beta testing measures included the Acceptability of Intervention Measure (AIM; *The My PreHab Program is appealing to me*), Intervention Appropriateness Measure (IAM; *The My PreHab Program seems suitable*) and Feasibility of Intervention Measure (FIM; *The My PreHab Program seems doable*) (17). These questions were measured on a 5-point Likert scale and assigned values ranging from 1 to 5 (1 = completely disagree; 2 = disagree; 3 = neither agree nor disagree; 4 = agree; 5 = completely agree). Scores were averaged, with higher scored indicating greater levels of acceptability, appropriateness, or feasibility (16). These measures were incorporated into the pathway as a patient experience survey sent to participants 2 weeks post registration.

## 3. Results

### 3.1. Phase 1: Collaborative codesign of *My PreHab* website

The *My PreHab* website (www.calhn-prehab.sa.gov.au) was designed to be a trusted resource for both consumers and their primary healthcare providers. The *My PreHab* website was designed as a stand-alone, publicly available, free resource, as well as incorporated into the digital *My PreHab Pathway*. The website provided information relating to nine prehab focus areas and their impact on the risk of preventable post-operative complications. These included: smoking, diabetes management, anaemia/low iron, emotional wellbeing, frailty, pain management, alcohol-drugs-medication management, activity and exercise, and nutrition and weight optimisation. Information was also provided on equipment and community services that might be needed post-operatively, to enable optimal preparation. Resources, service options or listings and relevant links were provided for both healthcare providers and consumers. Information from this website was incorporated into the *My PreHab Program* and provided as tailored targeted information to consumers in response to relevant prehab areas identified for them through a health screening questionnaire.

### 3.2. Phase 2: Understanding the needs and perspectives of consumers and primary healthcare providers

#### 3.2.1. The consumer perspective

Four major themes emerged from the consumer focus groups: the importance of being fit for surgery, having enough time to prepare, the need to be fully informed, and the trusted role of regular doctors.

##### 3.2.1.1. Theme 1: The importance of being physically and mentally fit for surgery

People who had recently undergone surgery highlighted the importance of both the body and mind being as prepared as possible prior to surgery to recover well.

*“Your body and mind need to be healthy”*

*“It's 100% important because you are going to get a quicker recovery”*

Most had adopted a ‘do-it-yourself’ approach to preparing for surgery and used the internet without support to try and identify how they could prepare for surgery. Important areas of health identified included: exercise and fitness, diet and nutrition, physical and mental strength.

Participants also wanted more information about potential complications from surgery and understanding ‘normal levels of pain’ and when to seek help.

Preparing the home environment and any equipment needed was also seen as essential.

##### 3.2.1.2. Theme 2: The importance of being fully informed

Focus group participants highlighted the need to be equipped with as much information as possible.

*“Being informed is very helpful”*

Many had discovered the importance of getting healthy before surgery from their previous surgical experiences and felt they had not been supported to prepare for surgery.

*“No-one provides this information”*

*“I feel like I have to work it out on my own, and get conflicting information from clinicians”*

*“There isn’t anyone to give you a look at the whole journey – pre, post, physical, mental, environment etcetera”*

*“It would be good if this was discussed before your first surgery”*

##### 3.2.1.3. Theme 3: Having enough time to prepare for surgery well

All participants (except for one who had received immediate surgery in the private health system) expressed the importance of being provided enough time to optimise their health and prepare well for surgery. They wanted this information as early as possible in their surgical journey. This was particularly important in terms of ‘getting into the system’ and arranging My Aged Care assessments so that health and community services could be accessed quickly when needed.

“*You need to be prepared as early as possible”*

*“They need to give us time to prepare….whether that be getting fit physically or mentally, or getting the house ready”*

##### 3.2.1.4. Theme 4: The trusted role of their regular doctor

Participants indicated that their regular doctors (GP) were trusted and best placed to provide information about prehab and guide them in their preparation.

*“It would be good to get general info (about how to prepare for surgery) from the GP initially, followed by more specific information about the surgery from the surgeon”*

*“My GP knows me best and could go through the whole journey with me”*

#### 3.2.2. Primary healthcare providers perspective

Three main themes emerged from discussions with primary care providers, acknowledging the importance of prehab and a team approach to supporting consumers, the lever surgery provides as a motivator for lifestyle change and that surgery is not always the solution. Additional barriers and enablers were also identified (Table 1), and changes made to the *My PreHab Pathway* to leverage identified enablers and reduce barriers (Supplementary Appendix C).

**Table 1:** Barriers and enablers to prehabilitation as identified by primary healthcare providers.

Barriers
GPs not knowing whether the consumers need surgery or not
Not knowing the timeframe to be seen for assessment or for surgery
Lack of two-way communication between primary care and hospital clinicians
Lack of knowledge about the ‘process’ once referred
Limited access to affordable allied health services
Enablers
Providing a realistic idea of the timeframe
Providing GPs and consumers the same information about what the surgery is and what to expect as early as possible
Phone clinic to discuss whether referral for surgery is needed
Making it a requirement of having surgery that consumers have engaged with their GP about prehab
Better remuneration for GPs to address lifestyle issues (time consuming/difficult to coordinate)
Funding for allied health services specifically for prehab (many consumers have used allocated care plans on other conditions)
Medicare item number for General Practice Nurse involvement
Providing information about affordable prehab services and actual options that can be accessed within the public health system in a timely manner
Providing GPs access to evidence of each prehab intervention in relation to types of surgery
Development of a handout to facilitate communication between the Hospital and the patient's GP regarding specific prehab requirements for each patient and goals that need to be met for surgery
Ongoing interface or engagement to check behavioural changes (during prehab)
Providing a pathway/program that enables patient engagement and “activation” not just knowledge

##### 3.2.1.5. Theme 1: Prehab is a team effort

It was widely acknowledged that although supporting healthy lifestyle changes is the remit of primary care there is scope to do it better prior to surgery. There was strong support for the best outcomes occurring where integrated care was provided by generalist and non-generalist clinicians working together (e.g., GPs and surgeons).

“*This is totally our raison d'etre”*

*“I’d be surprised if the GPs hadn’t ever given them lifestyle advice, but not surprised they were not given specific pre surgery advice. I don’t think we do this often”*

*“The GP is the co-ordinator, but an entire team is needed”*

*“Consumers always listen to and believe the specialists over their GP, but if they have a good relationship with their GP, we can work alongside them once they have committed to their lifestyle/health change”*

##### 3.2.1.6. Theme 2: Surgery as a motivational lever for positive lifestyle change

Primary care health providers were supportive of a prehab program and saw ‘anticipated’ surgery as a timely opportunity to activate their consumers to engage in positive lifestyle changes, that may in face negate the need for surgery.

“*Good concept, opportunity to implement health and advice and use surgery as the trigger”*

*I like the idea….using sur*gery *as an incentive - we can work together on that”*

*“…. and the changes might become permanent”*

“*I think the surgery hook is ideal to get patient buy in for the lifestyle changes. It is pretty rare for a patient to book an appointment with us just to talk about lifestyle issues so we will need to drive this. If it becomes a requirement of having surgery that they have engaged with their GP- perhaps this might work”*

##### 3.2.1.7. Theme 3: Surgery not always the answer

It was clear that many people referred for surgical assessment carried the unrealistic expectation that they would receive surgery, and conversations around the role and/or necessity for surgery were important to have.

“*Surgery does not always solve the issue….”*

*“Sometimes effective prehab obviates the need for surgery”*

*“There perhaps needs to be education for GPs and consumers/society that tests, and surgery are not necessarily the answer”*

### 3.2.1.8. Phase 3: Design and testing of *My PreHab Program* in orthopaedic surgery

#### 3.2.3. Design

The *My PreHab Pathway* is a text message mediated digital pathway that was created to screen consumers for potentially modifiable risk factors that can be addressed through prehab. It provides an individual summary report and targeted, evidence-based information and resources (housed on the *My PreHab* website) that consumers can use with their GP to develop a prehab plan that will work for them.

Upon responding to a text message invitation to register for the *My PreHab Pathway*, participants were welcomed and provided information about the wait time. Emphasis was placed on the opportunity to use the likely long wait to get as healthy as possible before surgery (prehab concept) to recover more quickly and minimise personal risk of complications. Participants were encouraged to complete a prehab health screening questionnaire and download a summary report to discuss with their GP. They also received information targeted to their personal risk factors from the *My PreHab* website along with advice as to how they might address these before surgery.

Eligible consumers were invited to register *via* text message. Non-responders received automatic text reminders. A list of registered consumers was available for clinical and administrative staff to view in the form of a “dashboard”. The dashboard also highlighted if a participant had completed their health screen and if there were any “red flags” for the nurses to review. The information collected in the screening process provided an opportunity for the nurses to review additional information about participants which may not have been declared in the referral. This assisted in more accurate triage and supported informed communication with the referring doctor especially where a referred consumer did not meet referral criteria or had significant risks.

#### 3.2.4. Testing

Testing occurred in two rounds. The first round (user-testing) was conducted in 30 participants and focussed on testing the functionality of the digital pathway, and the second round (beta-testing) was conducted over the first month of use in routine clinical practice (July 2022).

*User testing: Functionality*

**Figure 5:**
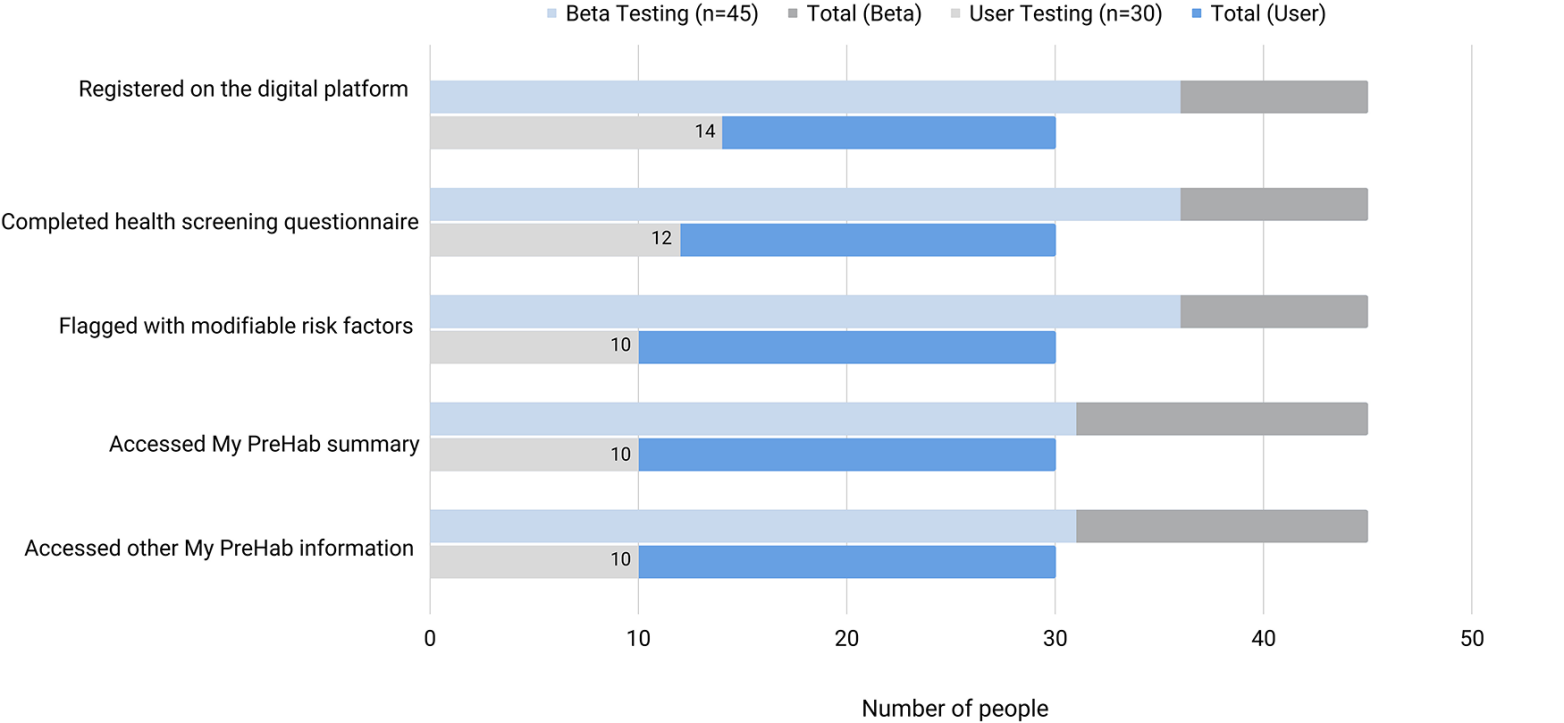
Consumer engagement with components of the *My PreHab Pathway* during user-testing and beta-testing in orthopaedic surgery.

Of the 30 consumers invited *via* text message, 14 registered on *My PreHab Pathway* [3 (50–59y); 5 (60–69y); 4 (70–79y) 8 (80–89y)]. Twelve out of 14 participants completed the health screening questionnaire, and 10 were found to have modifiable risk factors for post-operative complications (Figure 5). The majority indicating some level of confidence in being able to make a change (0 ‘very confident’, 1 ‘fairly confident’, 4 ‘somewhat confident’, 3 ‘slightly confident, 2 ‘not at all confident’). Paradoxically, those ‘not at all confident’ also wanted ‘little or no support’. The majority wanted ‘a solid plan of what to do’ (medium support, *n* = 6) and two wanted ‘some one-on-one assistance in some areas as well as a plan’ (a lot of support). Several target areas were identified for improvement and the *My PreHab Pathway* was modified accordingly (Table 2).

**Table 2:** Modifications made to the *My preHab pathway* after initial user testing.

Target	*My PreHab* Modification
Registrations on digital platform	Personalised invitation SMS to register
	Strengthened official branding
	Add additional reminders on day 3 and 7 (as well as 1)
Downloading *My PreHab* Summary Report	Added instructions
Scheduling appointments to discuss with GP	Added a digital survey prompt at 1 week to ascertain ‘intent to discuss *My Prehab* Summary Report’ with GP.
Sustained engagement with lifestyle modifications	Added prehab re-assessment every 6 months on wait list.

*Beta-Testing*

Within the first month of implementation (beta-testing) of the modified pathway 66 consumers were triaged as category two or three on the outpatient waitlist for orthopaedic assessment. Twenty-one were excluded (11 no personal mobile number; 1 incorrect mobile number; 2 shared mobile; 1 repeat referral; 5 requiring interpreter, 1 human error). Forty-five were invited to register on the *My PreHab Pathway*.

During the first month of implementation people with a shared mobile phone and some who required an interpreter were found to have been excluded in error and have since been invited. However, they are excluded from initial Beta-testing results from July reported here.

**Figure 6:**
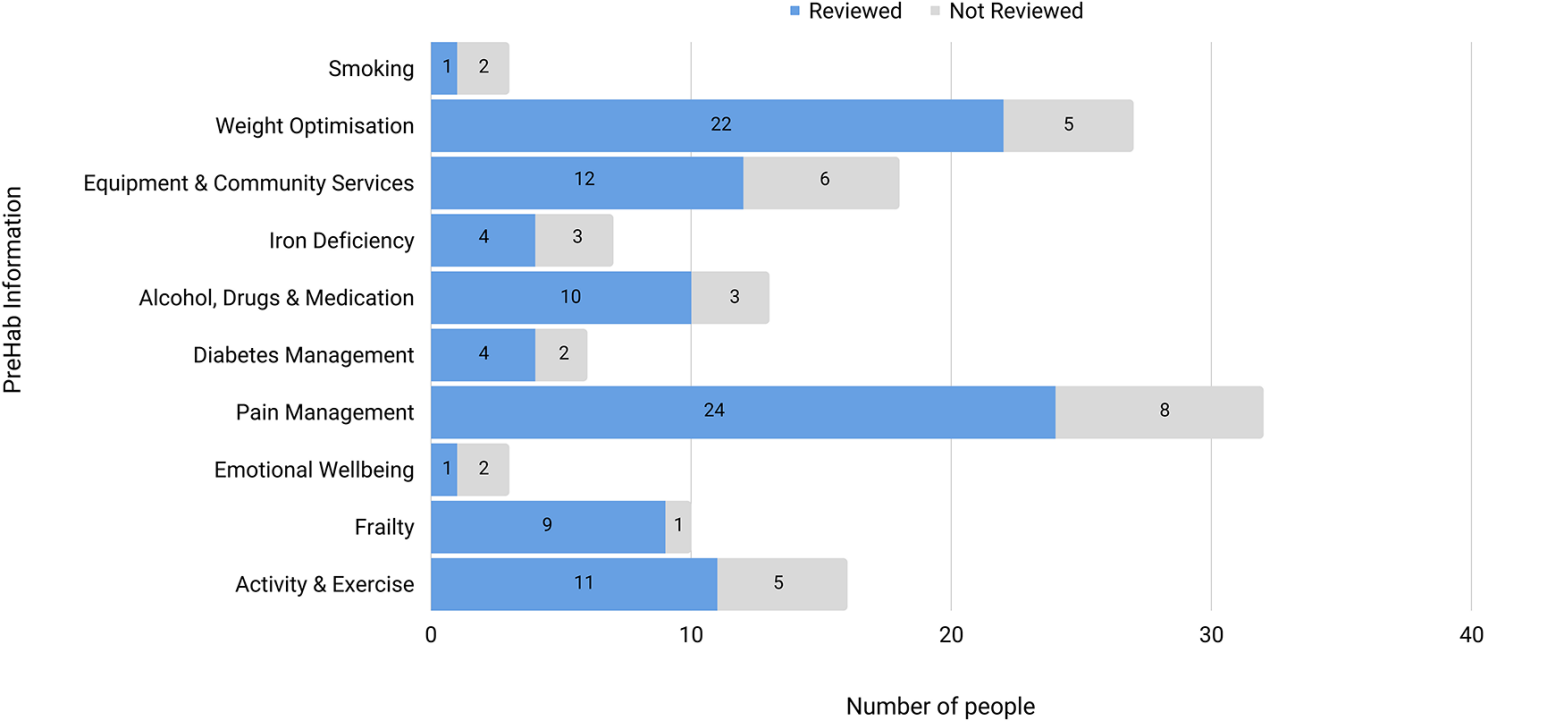
Prehab areas identified and the number of people who reviewed the information on each area.

A greater registration rate was seen with 36/45 (80%) registering compared with 14/30 (47%) during initial user testing. Respondents were aged between 45 and 85 [2 (45–49); 7 (50–59y); 16 (60–69y); 10 (70–79y); 1 (80–85y)], and four people had a proxy complete their health questionnaire (two required an interpreter as documented in their hospital electronic medical record). Non-respondents were aged between 58 and 77 years, and all spoke English as their first language. All participants completed the health-screening survey and were flagged with at least one modifiable risk factor for post-operative complications. The breakdown of prehab focus areas identified and proportion of registrants who reviewed the relevant *My PreHab* information is depicted in Figure 6.

Over half (*n* = 19) of the participants were categorised as ‘obese’ with a BMI > 30 (6 had BMI > 40). Screening of these results by the Joint Replacement Nurse Consultant and Practitioner resulted in seven warning letters being issued to GPs (regarding consumers with high BMIs or multiple risk factors) and the rejection of one referral (high BMI).

Half of the participants (*n* = 18) completed the 2-week post-registration consumer experience questionnaire. Of these, five had already seen their GP, six had scheduled an appointment, five planned to schedule an appointment and two did not plan to follow up with their GP. Of these, ten had started working on improving prehab target areas, seven planned to, and one did not plan to. Half of the respondents indicated they were likely (*n* = 7) or very likely (*n* = 2) to recommend *My PreHab* to others {neither likely or unlikely, *n* = 6; unlikely, *n* = 3} 6 were (Figure 4). The *My PreHab Program* scored an average 3.4 (SD 0.78) for acceptability, 3.5 (SD 0.62) for appropriateness, and 3.6 (SD 0.61) for feasibility.

## 4. Discussion

This study shows that a proportion of consumers and GPs recognise the value of prehab in reducing the risk of postoperative complications and maximising recovery from planned (elective) surgery. With minimal cost and effort, 80% of people referred for hip/knee surgery assessment registered for the pathway, completed a health screening questionnaire, and accessed information relating to prehab. The majority of respondents at 2-week follow up intended to discuss their *My PreHab* report with their GP or had already done so.

Clearly, not all consumers or GPs will be as invested, however, any shift towards mutual holding of responsibility for the outcomes of surgery is likely to be a good thing and yield a reduction in the rate of preventable post-operative complications. The clinical and health-system impact of *My Prehab Program* is currently being assessed in a hybrid implementation-effectiveness trial however as surgical wait-times are long there is value to be gained in these early data. We anticipate that supporting even a portion of consumers to address modifiable risk factors before surgery and reduce their risk of complications, would free up more resources for those who are unable to do so, yielding measurable system level benefits.

Many aspects of prehab simply reflect good routine care (weight, smoking, anaemia, diabetes, mental health) and are always encouraged, forming the backbone of GP care. Based on our consultations, we anticipate GPs to gain benefit from having all these resources being in one place and that the lever the screening tool results provide for conversation and patient engagement in lifestyle changes. There is likely to be a proportion of people who need additional support to implement lifestyle changes, however we have listed and/or linked to a variety of referral resources that GPs can use. *My PreHab Program* has not been designed to be all things to all people, however early data suggest it is broadly acceptable, feasible, and valued by staff and consumers. Based on current behaviour of people who have engaged with the program, it is likely to have bigger “ripple out” effects as alignment in the expectations of hospitals, referring doctors, and the community occurs.

The *My PreHab* website has been made publicly available and there appears to be considerable interest. During July 2022 there were 190 visitors to the site over 1,037 sessions (Google Analytics). This activity is well above that expected from 45 study participants. A number of surgical units across Australia have also enquired about using the *My PreHab Program* for their patients. There is considerable opportunity to scale the *My PreHab Program* to other surgical areas, and we are currently developing digital *My PreHab* Pathways for General Surgery (including complex hernias, rectal cancer, bariatrics).

The *My PreHab Program* aligns with the following principles developed to guide improvement in perioperative care in Australia (18).

• All planning should be based around the consumer, their expectations and needs; and is informed through an evidence base and individualised to the patient's risk profile
• Modifiable risk should be identified and addressed as early as possible, preferably within the primary care setting and with consumer buy-in.
• The pathway for preventing surgical complications starts with primary care.
• Decentralised preventative care using telehealth and community-based care is essential and where possible managed in the consumer's home and other out-of-hospital settings (e.g., exercise, diet, smoking cessation etc).
• Care pathways should be designed using the best available evidence and should minimise unnecessary variation and maximise consistency.
• Evidence-based approaches should be used within all elements of the system, gaps should be identified and addressed in a structured manner.
• There is clear communication and accountability throughout the perioperative care journey.
• A model of shared decision making should be embraced by clinicians and involve consumers, and their carers and family, and information should be readily accessible to all stakeholders.

*My PreHab Program* is a novel, pragmatic approach that if successful, is highly likely to yield significant health gains not only to people undergoing surgery, (and those for whom surgery may be no longer needed due) but also to others reaping the benefits of a heightened GP and community awareness of health across Australia. The *My PreHab Program* is both cost and resource efficient with potential to easily scale up across medical specialties within local jurisdictions as well as state-wide and nationally where evidence-based guidelines exist. This could improve equity of access to care and optimise outcomes minimising the “postcode lottery” we currently have in Australia, and it could minimise unwarranted variation which is an important principle in the delivery of high value care.

## Data Availability

The raw data supporting the conclusions of this article will be made available by the authors, without undue reservation.
