# Supplementary material for: Development of a feasible and acceptable digital prehabilitation pathway to improve elective surgical outcomes
Source: Front Digit Health. 2023 Feb 9;5:1054894. doi: 10.3389/fdgth.2023.1054894 (PMC9947781; doi:10.3389/fdgth.2023.1054894)
Supplement: Supplementary file 1 [file Table1.docx]

**Appendix A: *Using the Theoretical Domains Framework to develop focus group questions for primary healthcare providers***

| Domain | Example Question |
| --- | --- |
| Knowledge | What is your understanding of the importance of optimising a consumers’ health prior to surgery (prehab)? |
| Skills | Can you think of any way your own skills in optimising a consumers’ health prior to surgery could be improved? |
| Social/professional role and identity | To what extent do you consider optimising a patient’s health prior to surgery part of your role? |
| Beliefs about capabilities | Are there any aspects of prehab that you feel more or less comfortable performing? OR  How confident are you that you can optimise a patient’s health before surgery? |
| Optimism | How optimistic or pessimistic are you that optimising patient care prior to surgery holds the potential to improve the outcomes of consumers you refer for planned surgery? |
| Beliefs about consequences | To what extent do you believe optimising patient care prior to surgery will improve the outcomes of consumers you refer for planned surgery? |
| Reinforcement | Are you aware of any ways in which optimising patient health prior to surgery is rewarded? |
| Intentions | To what extent do you intend to (or continue to) optimise the health of consumers referred for planned surgery? |
| Motivations and goals | What motivates you to optimise the health of consumers referred for planned surgery (or not to)? OR  Are there other things you want or need to do during your practice that prevent optimising patient health prior to surgery? |
| Memory, attention, and decision processes | How easy or difficult it is to initiate health preoptimization discussions and processes following referral for consideration of surgery? |
| Environmental context and resources | What factors help or hinder your ability to optimise patient health prior to surgery?    To what extent do you have sufficient resources and supports to optimise patient health prior to surgery? |
| Social influences | Are there any conflicting beliefs amongst your colleagues as to the importance/value of prehab? |
| Emotions | How do you feel when you think about optimising your consumers’ health prior to surgery? |
| Behavioural regulation | Are there any systems in place to monitor the optimisation of patient health prior to surgery in your practice? |

**Appendix B: *Using the Theoretical Domains Framework to develop focus group questions for consumers***

| **Domain** | **Example Question** |
| --- | --- |
| Knowledge | Did you receive information about ‘getting healthy before surgery’ before your surgery?  Who provided this information and when?  Would you have liked further information?  How important do you think ‘getting healthy before surgery is and why’? |
| Skills | Do you feel you had the skills to find the information, advice or support needed to ‘get healthy before surgery’? |
| Social/professional role and identity | Do you think you have a role in deciding what you can do to reduce your risk in experiencing surgical complications that can be avoided? |
| Beliefs about capabilities | How confident are you in your ability to discuss and decide on a plan to improve your health before surgery with your GP? |
| Optimism | How optimistic are you that ‘getting healthy before surgery’ can prevent complications from surgery? |
| Beliefs about consequences | What do you think are the benefits and disadvantages of trying to improve your health before planned surgery? |
| Reinforcement | Are you aware of any ways in which getting healthy before surgery is rewarded? |
| Intentions | Did you plan to improve your health before surgery? |
| Motivations and goals | What stopped you (or would stop you in the future) from trying to improve your health before surgery? |
| Memory, attention and decision processes | N/A |
| Environmental context and resources | How well did you feel supported by your GP to improve your health before surgery? |
| Social influences | N/A |
| Emotions | How do you feel when you think about trying to improve your health before surgery? |
| Behavioural regulation | N/A |

***Appendix C: Modifications made to the My PreHab Pathway following consultation with primary care providers***

| **Enabler** | **Addressed in My PreHab Pathway** |
| --- | --- |
| Providing a realistic idea of the timeframe | Welcome message references the ‘long wait time’ |
| Providing GPs and consumers the same information as soon as possible | The website is publicly accessible and incorporates information for GPs and consumers |
| Making it a requirement of having surgery that consumers have engaged with their GP about prehab | It is not a ‘requirement’, however the digital *My PreHab Pathway* it has been built into ‘routine care’  There is also an incentive to complete the health assessment questionnaire “please complete the health questionnaire to assist us to progress your referral”. |
| Providing information about affordable prehab services and actual options that can be accessed within the public health system in a timely manner | Links to specific services (and service listings through HealthPathways SA) provided |
| Providing GPs access to evidence of each prehab intervention in relation to types of surgery | We have provided information and evidence on the impact of each prehab intervention on risk of post-operative complications in general, but not specific types of surgery. |
| Development of a handout to facilitate communication between the Hospital and the patient’s GP regarding specific prehab requirements for each patient and goals that need to be met for surgery | Individualised *My PreHab* Summary Report provided as a download for consumers to discuss with their GPs |
| Ongoing interface or engagement to check behavioural changes (during prehab) | Built in reminders and follow up activities throughout the wait time.   - reminder to see the GP at 1 week - 2-week check-in on progress - 6-month check-in - 6 monthly prompts |
